# Amyloid-Beta Induces Different Expression Pattern of Tissue Transglutaminase and Its Isoforms on Olfactory Ensheathing Cells: Modulatory Effect of Indicaxanthin

**DOI:** 10.3390/ijms22073388

**Published:** 2021-03-25

**Authors:** Agata Campisi, Giuseppina Raciti, Giovanni Sposito, Rosaria Grasso, Maria A. Chiacchio, Michela Spatuzza, Alessandro Attanzio, Ugo Chiacchio, Luisa Tesoriere, Mario Allegra, Rosalia Pellitteri

**Affiliations:** 1 Department of Drug Sciences and Health, University of Catania, 95125 Catania, Italy; racitigi@unict.it (G.R.); giovanni.sposito@hotmail.it (G.S.); ma.chiacchio@unict.it (M.A.C.); uchiacchio@unict.it (U.C.); 2 Department of Physics and Astronomy “Ettore Majorana”, University of Catania, 95123 Catania, Italy; grassosara018@gmail.com; 3 Institute for Biomedical Research and Innovation (IRIB), National Research Council, 95126 Catania, Italy; michela.spatuzza@cnr.it (M.S.); rosalia.pellitteri@cnr.it (R.P.); 4 Department of Biological, Chemical, and Pharmaceutical Sciences and Technologies (STEBICEF), University of Palermo, 90128 Palermo, Italy; alessandro.attanzio@unipa.it (A.A.); luisa.tesoriere@unipa.it (L.T.)

**Keywords:** tissue transglutaminase, olfactory ensheathing cells, amyloid-beta, oxidative stress, Indicaxanthin, self-renewal

## Abstract

Herein, we assessed the effect of full native peptide of amyloid-beta (Aβ) (1-42) and its fragments (25-35 and 35-25) on tissue transglutaminase (TG2) and its isoforms (TG2-Long and TG2-Short) expression levels on olfactory ensheathing cells (OECs). Vimentin and glial fibrillary acid protein (GFAP) were also studied. The effect of the pre-treatment with indicaxanthin from Opuntia ficus-indica fruit on TG2 expression levels and its isoforms, cell viability, total reactive oxygen species (ROS), superoxide anion (O_2_^−^), and apoptotic pathway activation was assessed. The levels of Nestin and cyclin D1 were also evaluated. Our findings highlight that OECs exposure to Aβ(1-42) and its fragments induced an increase in TG2 expression levels and a different expression pattern of its isoforms. Indicaxanthin pre-treatment reduced TG2 overexpression, modulating the expression of TG2 isoforms. It reduced total ROS and O_2_^−^ production, GFAP and Vimentin levels, inhibiting apoptotic pathway activation. It also induced an increase in the Nestin and cyclin D1 expression levels. Our data demonstrated that indicaxanthin pre-treatment stimulated OECs self-renewal through the reparative activity played by TG2. They also suggest that Aβ might modify TG2 conformation in OECs and that indicaxanthin pre-treatment might modulate TG2 conformation, stimulating neural regeneration in Alzheimer’s disease.

## 1. Introduction

Alzheimer’s disease (AD) is characterized by intracellular and extracellular protein aggregates in the brain including microtubule-associated protein tau and cleavage products of the amyloid precursor protein, amyloid-beta (Aβ). The accumulation of Aβ is responsible for oxidative stress, inflammation, and neurotoxicity, which lead to apoptosis and the deterioration of the neurotransmission system observed in AD [1]. Aβ is also a substrate for tissue transglutaminase (TG2), a ubiquitarian calcium-dependent protein that catalyzes cross-linking reactions, inducing Aβ oligomerization and aggregation, which are typical signs of AD [2]. It also has disulfide isomerase [3], kinase, and GTPase activities [4]. TG2 is mainly localized in the cytosol (73%), partially in the plasma membrane (20%), nucleus (7%), and extracellular matrix [5,6,7]. The functions of TG2 depend on its intracellular localization. When it is localized in the cytosol, TG2 controls apoptotic processes in a stimuli-dependent manner, through its transamidating activity that is necessary for pro-apoptotic effects [6]. Instead, when TG2 is localized into the nuclear compartment, it phosphorylates different proteins including retinoblastoma and p53, which are known to be substrates for TG2 kinase activity [8]. Furthermore, TG2 is present in two isoforms: short TG2 (TG2-S) and long TG2 (TG2-L), having different cellular localization that mediates opposite cellular functions [9,10,11]. In particular, TG2-S, localized in the cytosol and mitochondria, increases during apoptosis, which is responsible for the aggregate formation and involved in AD [9,10,11]. In contrast, TG2-L, localized in the nucleus, exerts a protective effect against cellular injury and apoptosis due to its transamidating activity [9,10,11].

Several findings have reported that in AD patients, an early sign of neurodegeneration is represented by a reduced function of olfactory performance [12]. In particular, a peculiar olfactory glial cell is represented by olfactory ensheathing cells (OECs). This cellular type, showing a bipolar or multipolar morphology, surrounds the olfactory nerves and is able to secrete different growth factors, neurotrophins, adhesion molecules, and numerous markers, which promote neuron survival and axonal growth, even supporting an injured central nervous system (CNS) [13,14,15,16]. OECs are able to stimulate angiogenesis and remyelination; therefore, they play an important role in transplants in spinal cord injury [17]. In addition, OECs exhibit stem cell properties, expressing Nestin, a marker of precursor neural stem cells [17]. In previous studies, we demonstrated that TG2 was overexpressed in OECs exposed to full native peptide Aβ(1-42) and Aβ(25-35) fragment and that some growth factors were able to downregulate the expression levels of the protein [18].

In recent years, growing attention rose on neuro-nutraceuticals such as indicaxanthin, a phytochemical produced by cactus pear fruit from *Opuntia ficus-indica*, L. Mill [19,20]. Indicaxanthin possesses significant anti-proliferative, antitumor, and anti-inflammatory effects both in vivo and in vitro [21,22]. In addition, it modulates the reactive oxygen species (ROS) production, prevents mitochondrial damage, regulates cell redox balance, and calcium homeostasis in several experimental in vitro models [23]. Interestingly enough, and in contrast with the majority of phytochemicals, indicaxanthin is able to cross the blood–brain barrier and to modulate the bioelectric neuronal activity in the hippocampus [24].

Herein, we assessed TG2, TG2-S, and TG2-L expression levels in OECs exposed to Aβ(1-42) or Aβ(25-35) or reverse-sequence fragment Aβ(35-25) [25] and the effect of indicaxanthin. The expression levels of some cytoskeletal proteins such as Vimentin, a marker of gliosis and also a substrate of TG2, glial fibrillary acid protein (GFAP), a glial marker of growth, maturation, and differentiation, were evaluated. Since Nestin, a marker of neural precursors, is co-expressed in pluripotent stem cells with cyclin D_1_ [26], a marker of cellular proliferation, the effect of indicaxanthin pre-treatment on their levels was tested. Furthermore, its effect on cell viability, on the production of total reactive oxygen species (ROS) and superoxide anion (O_2_^−^) and on apoptotic pathway activation was assessed.

## 2. Results

### 2.1. Cell Viability

**Figure 1:**
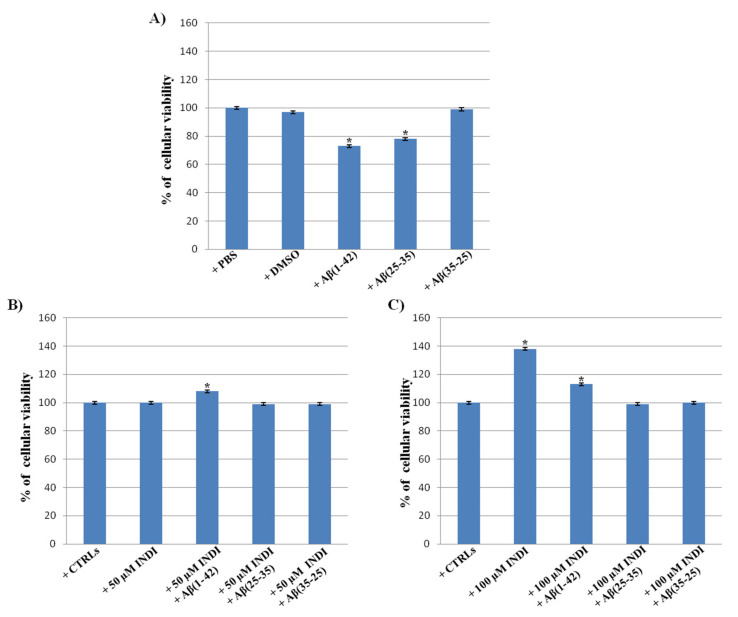
Percentage of cell viability in Olfactory Ensheating Cells (OECs) performed through the 3(4,5-dimethyl-thiazol-2-yl)2,5-diphenyl-tetrazolium bromide (MTT) test. (**A**) Phosfate buffer saline (PBS) and dimethyl sulfoxide (DMSO) treated OECs, exposed to 10 μM Amyloid Beta (Aβ) Aβ (1-42) or Aβ(25-35) or Aβ(35-25) for 24 h; (**B**) OECs pre-treated with 50 µM indicaxanthin (INDI) and exposed to 10 μM Aβ(1-42) or Aβ(25-35) or Aβ(35-25) for 24 h; (**C**) OECs pre-treated with 100 µM INDI and exposed to 10 μM Aβ(1-42) or Aβ(25-35) or Aβ(35-25) for 24 h. Data were statistically analyzed by using one-way analysis of variance (one-way ANOVA) followed by post-hoc Holm–Sidak test, in order to calculate significant differences among groups. Data reported represent the mean ± S.D. of five separated experiments in triplicate. * p < 0.05 significant differences vs. controls.

To monitor cell viability in OECs unexposed and exposed to full native peptide Aβ(1-42) or to the fragments Aβ(25-35) and Aβ(35-25) [18], both in the absence and in the presence of indicaxanthin, the 3(4,5-dimethyl-thiazol-2-yl)2,5-diphenyl-tetrazolium bromide (MTT) test was performed. In our previous studies, we showed that the optimal concentration of Aβ(1-42), Aβ(25-35), and Aβ(35-25) was 10 μM for 24 h [18]. No significant differences between PBS and DMSO-treated OECs were observed, thus they were used as controls. A significant decrease in the percentage of cell viability in OECs exposed to 10 μM Aβ(1-42) or Aβ(25-35) was found when compared with the controls (Figure 1A). The reverse sequence of Aβ(35-25) did not show any effect on cell viability when compared with the controls (Figure 1A). No significant change in the percentage cell viability was observed in OECs exposed to 50 µM (Figure 1B) of indicaxanthin when compared with the controls, while a significant increase was found with 100 µM (Figure 1C). The treatment of OECs with 100 µM of indicaxanthin was more effective compared with 50 µM; therefore, we chose 100 µM as the optimal concentration. The pre-treatment of OECs with 50 µM and 100 µM of indicaxanthin and the subsequent exposure to 10 µM Aβ(1-42) or Aβ(25-35) for 24 h was able to restore cell viability to the levels observed in the controls (Figure 1B,C). Indicaxanthin pre-treatment did not induce any significant changes in OECs exposed to the Aβ(35-25) fragment.

Thus, for the following studies, OECs were pre-treated with 100 μM of indicaxanthin for 30 min and subsequently exposed to native peptide 10 μM Aβ(1-42) or to the fragments Aβ(25-35) or Aβ(35-25) for 24 h.

### 2.2. Vimentin and Glial Fibrillary Acid Protein (GFAP) Immunolabeling

**Figure 2:**
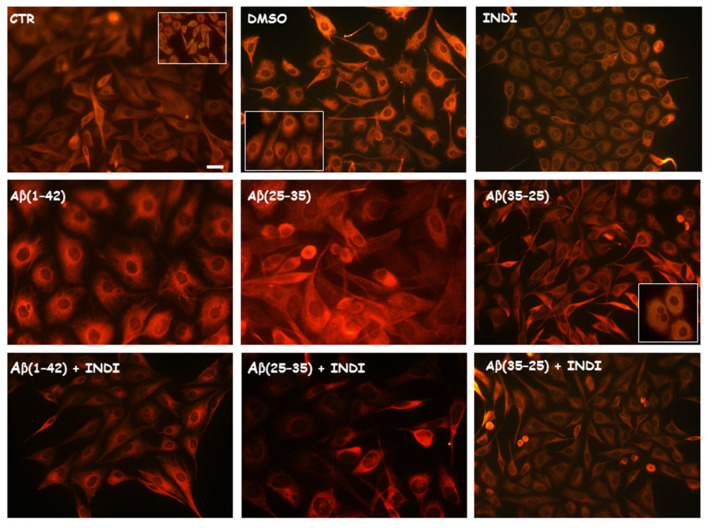
Immunocytochemistry for anti-Vimentin in OECs. Images show different conditions: control, DMSO, 100 µM Indicaxanthin (INDI), 10 μM Aβ(1-42) or Aβ(25-35) or Aβ(35-25) both in the absence and presence of 100 µM indicaxanthin for 24 h. Scale bar 20 µm.

**Figure 3:**
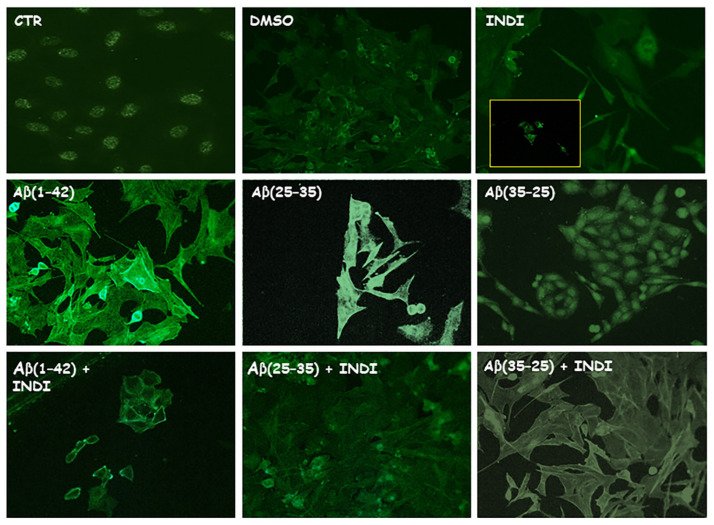
Immunocytochemistry for anti-GFAP in OECs. Images show different conditions: control, DMSO, 100 µM Indicaxanthin (INDI), 10 μM Aβ(1-42) or Aβ(25-35) or Aβ(35-25) both in the absence and presence of 100 µM INDI for 24 h. Scale bar 20 µm.

To identify glial reactivity after the treatment of OECs exposed to the full native peptide Aβ(1-42) or to Aβ(25-35) or Aβ(35-25) fragments both in the absence and in the presence of indicaxanthin, we performed immunostaining using antibodies against cytoskeletal proteins Vimentin and GFAP. The exposure of the cells to Aβ(1-42) or Aβ(25-35) was able to induce a notable increase in the number of cells positive to Vimentin (Figure 2) and GFAP (Figure 3), when compared with the control cultures and those treated with the fragment Aβ(35–35). In particular, the effect appeared more evident in Aβ(25-35) exposed OECs when compared with Aβ(1-42) ones. Aβ (1-42 and 25-35) exposure provoked an increase in cell size, as shown in Figure 2 and Figure 3. The treatment of cultures with 100 μM of indicaxanthin did not induce changes for the positivity of the cells for Vimentin and GFAP when compared with the controls. When the cells were pre-treated with indicaxanthin and then exposed to Aβ(1-42) or Aβ(25-35), the cell positivity for Vimentin and GFAP appeared similar to those observed in the control cultures. No specific staining of OECs was observed in control incubation in which the primary antibodies were omitted. This group of experiments demonstrated that full native peptide Aβ(1-42) and fragment Aβ(25-35) induced glial activation and that indicaxanthin pre-treatment was able to counteract it.

### 2.3. Total Reactive Oxygen Species (ROS)/O_2_^−^ Generation

**Figure 4:**
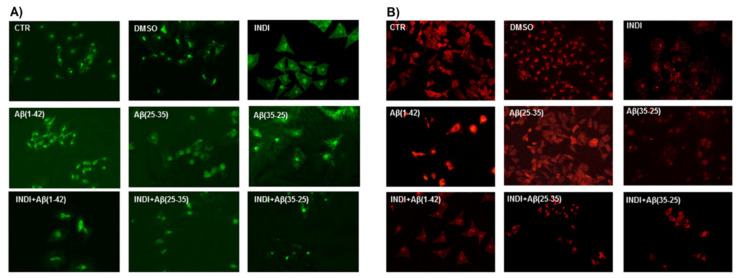
Staining of total intracellular reactive oxygen species (ROS) levels and O_2_^−^. Total intracellular ROS levels (**A**) and O_2_^−^ (**B**) generation in OECs in different conditions: control, DMSO, 100 µM indicaxanthin (INDI), 10 μM Aβ(1-42) or Aβ(25-35 ) or Aβ(35-25) both in the absence and presence of 100 µM INDI for 24 h. Scale bar 20 µm.

To monitor the intracellular oxidative status, the staining of total intracellular ROS levels (Figure 4A, green) and O_2_^−^ (Figure 4B, red) generation in OECs exposed for 24 h to the full native peptide Aβ(1-42) or to the fragment Aβ(25-35) or to Aβ (35-25), both in the absence and presence of indicaxanthin, was assessed. Aβ treatment induced a significant increase in total ROS and O_2_^−^ levels when compared with the controls. No significant change was found in total ROS and O_2_^−^ production in Aβ(35-25) and indicaxanthin alone exposed cells. The OEC pre-treatment with indicaxanthin and the subsequent exposure to Aβ(1-42) or to Aβ(25-35) or to Aβ(35-25) induced a strong reduction of both the total intracellular ROS and O_2_^−^ levels. These findings highlighted that Aβ(1-42) and Aβ(25-35) increased the levels of total ROS and O_2_^−^ production and that indicaxanthin pre-treatment was able to restore the oxidative status modified by Aβ to the control values, reducing prevalently O_2_^−^ generation.

### 2.4. Total TG2 Expression through Immunocytochemistry

**Figure 5:**
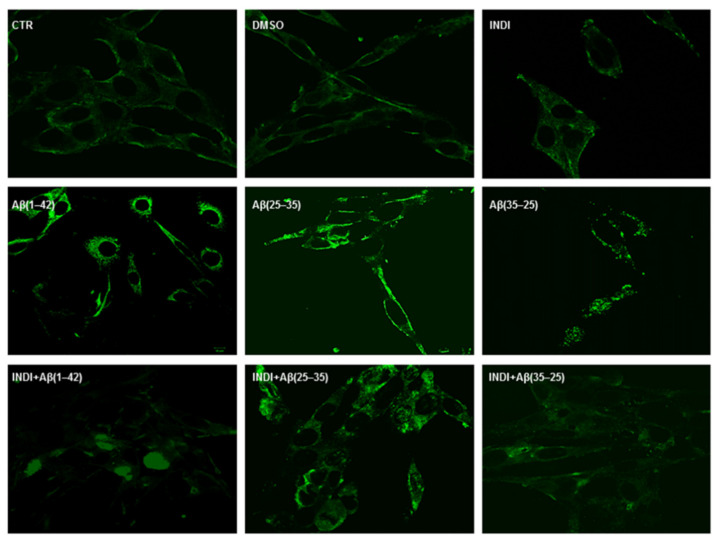
Confocal laser scanning microscopy of labeling immunocytochemistry for anti-TG2 in OECs. Images show different conditions: control, DMSO, 100 µM indicaxanthin (INDI), 10 μM Aβ(1-42) or Aβ(25-35) or Aβ(35-25) both in the absence and presence of 100 µM INDI for 24 h. Scale bar 20 µm.

Figure 5 reports TG2 positivity and its localization performed on single cells through immunocytochemical procedures and confocal laser scanning microscope (CLSM) analysis. In the control cells, a low staining for TG2 was found and the protein was prevalently localized in the cytosol. A more intense staining for TG2 both in Aβ(1-42) and Aβ(25-35) treated OECs was observed widely in the cytosol when compared with the controls. In particular, the positivity of the cells for TG2 appeared more evident in the Aβ(25-35) treated ones when compared with the controls and Aβ(1-42) treated OECs. The exposure to the fragment Aβ(35-25) produced a slight increase for TG2 cell positivity when compared with the controls and the protein appeared prevalently localized in the cytosol. In 100 μM indicaxanthin-treated cells, TG2 staining was slightly higher than controls and it was widely localized in the cytosol. In contrast, in the cells exposed to Aβ(1-42) and Aβ(25-35 ), indicaxanthin pre-treatment was able to decrease the number of positive cells for TG2 when compared with the controls. Specifically, in indicaxanthin treated OECs and subsequently exposed to Aβ(1-42), TG2 was localized into the nucleus. Unlike in Aβ(25-35)-indicaxanthin pre-treated cells, the protein was prevalently localized in the cytosol, even if some cells showed a low positivity for the protein in the nucleus and nucleoli. A low staining for TG2 in indicaxanthin pre-treated OECs and then exposed to the fragment Aβ(35-25) was found when comparing both with the controls and the fragment Aβ(35-25) alone. In addition, the protein was prevalently localized in the cytosol. No specific staining of OECs was observed in control incubation in which the primary antibody was omitted.

### 2.5. TG2 and Its Isoform Expression and Effect of Indicaxanthin

**Figure 6:**
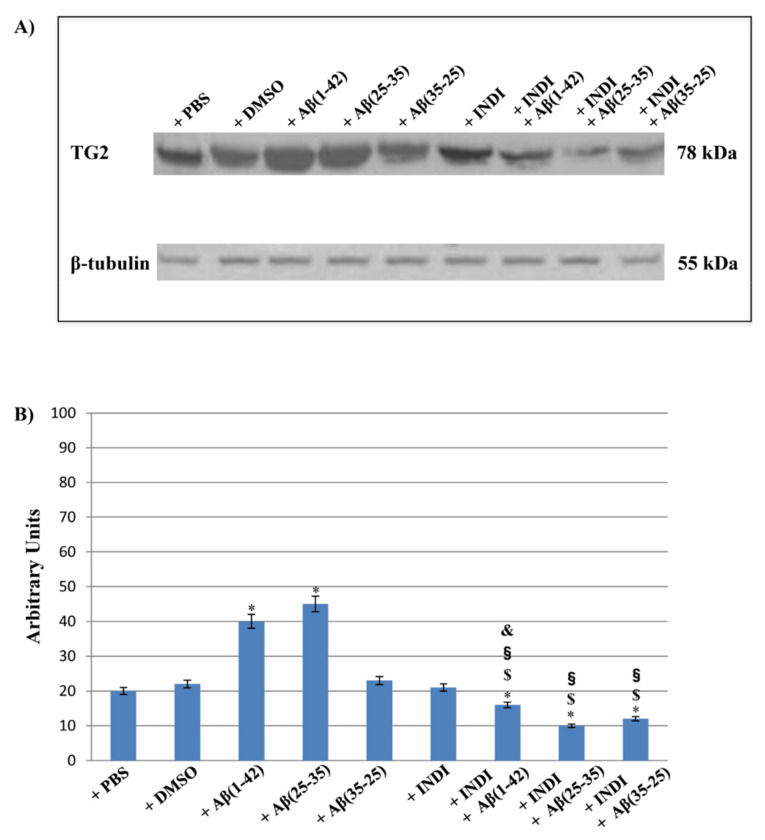
Western blotting analysis. (**A**) Representative immunoblot for total tissue transglutaminase (TG2) expression levels in total cellular lysates from OECs in different conditions: control, DMSO, 100 µM indicaxanthin (INDI), 10 μM Aβ(1-42) or Aβ(25-35) or Aβ(35-25) both in the absence and presence of 100 µM INDI for 24 h. (**B**) Densitometric analysis of TG2 expression levels performed after normalization with β-tubulin. The results are expressed as the mean ± S.D. of the values of five separate experiments performed in triplicate. * *p* < 0.05 significant differences vs. controls; **^$^** *p* < 0.05 significant differences of Aβ(1-42) + INDI or Aβ(25-35) + INDI or Aβ(35-25) + INDI vs. INDI; **^§^** *p* < 0.05 significant differences of Aβ(1-42) + INDI vs. Aβ(1-42), Aβ(25-35) + INDI vs. Aβ(25-35) or Aβ(35-25) + INDI vs. Aβ(35-25); **^&^** *p* < 0.05 significant differences of Aβ(1-42) + INDI vs. Aβ(25-35) + INDI or vs. Aβ(35-25) + INDI.

To assess and better clarify CLMS analysis performed on single cell relative to the different intracellular localization of TG2, the expression levels of the total TG2, and its isoforms (TG2-S and TG2-L) induced by the different treatment types were evaluated through western blot analysis on total cellular lysates. Immunoblots (Figure 6A) and densitometric analysis (Figure 6B) showed a significant increase in total TG2 expression levels in both Aβ(1-42) and Aβ(25-35) exposed OECs when compared with the controls. The effect was more evident in Aβ(25-35)-treated cells. Slight but no significant changes in total TG2 expression levels in cultures exposed to Aβ(35-25) were found. Indicaxanthin pre-treatment did not induce significant modifications in total TG2 expression levels when compared with the controls. When indicaxanthin was added to OECs following exposure to Aβ(1-42) or Aβ(25-35) or Aβ(35-25), a significant reduction of total TG2 expression levels was observed when compared with the controls. The effect of the pre-treatment of the cells with indicaxanthin was more evident in OECs exposed to Aβ(25-35). In indicaxanthin pre-treated OECs then exposed to the fragment Aβ(35-25), a significant reduction in total TG2 expression levels was relieved when compared with Aβ(35-25), indicaxanthin alone treated cells, and with the controls.

**Figure 7:**
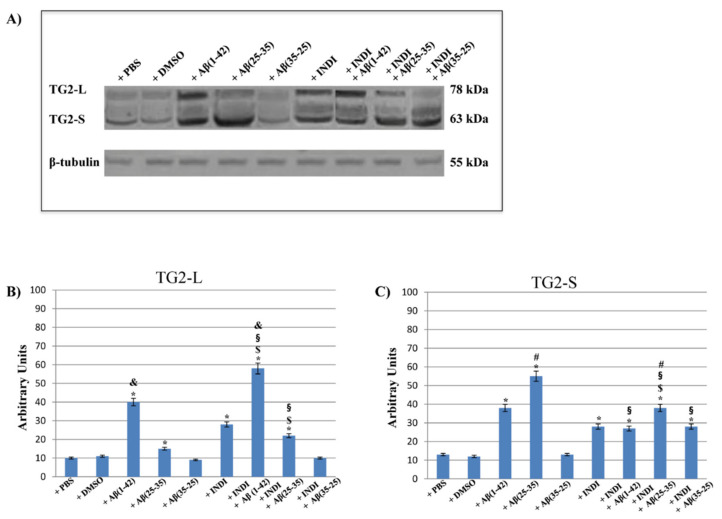
Western blotting analysis. (**A**) Representative immunoblots through western blotting analysis for TG2 isoform (tissue transglutaminase long, TG2-L; tissue transglutaminase short, TG2-S) expression levels in total cellular lysate from OECs in different conditions: control, DMSO, 100 µM Indicaxanthin (INDI), 10 μM Aβ(1-42) or Aβ(25-35) or Aβ(35-25) both in the absence and presence of 100 µM INDI for 24 h; (**B**) Densitometric analysis of TG2-L expression levels performed after normalization with β-tubulin. (**C**) Densitometric analysis of TG2-S expression levels performed after normalization with β-tubulin. The results are expressed as the mean ± S.D. of the values of five separate experiments performed in triplicate. * *p* < 0.05 significant differences vs. controls; **^$^** *p* < 0.05 significant differences of Aβ(1-42) + INDI or Aβ(25-35) + INDI or Aβ(35-25) + INDI vs. INDI; **^§^** *p* < 0.05 significant differences of Aβ(1-42) + INDI vs. Aβ(1-42), Aβ(25-35 ) + INDI vs. Aβ(25-35) or Aβ(35-25) + INDI vs. Aβ(35-25); **^&^** *p* < 0.05 significant differences of Aβ(1-42) vs. Aβ(25-35) or vs. Aβ(35-25) and of Aβ(1-42) vs. Aβ(25-35) + INDI or vs. Aβ(35-25) + INDI; **^#^** *p* < 0.05 significant differences of Aβ(25-35) vs. Aβ(1-42) or vs. Aβ(35-25) and of Aβ(25-35) + INDI vs. Aβ(1-42) + INDI or vs. Aβ(35-25) + INDI.

To better elucidate the effect of OEC exposure to Aβ(1-42) or Aβ(25-35) or Aβ(35-25) both in the absence and presence of indicaxanthin on the role played by TG2, the expression levels of its isoforms were detected. Figure 7 reports the immunoblots (Figure 7A) and densitometric analysis (Figure 7B,C) performed on all experimental conditions. Based on the treatment type, different expression patterns of the isoforms were found. In the controls (PBS and DMSO), both TG2 isoforms were expressed at very low levels, even if TG2-S levels were higher than TG2-L. Aβ(1-42) treatment induced a significant increase in both isoform expression levels when compared with the controls. The exposure of OECs to Aβ(25-35) caused a significant increase of TG2-S and TG2-L, even if TG2-S levels were higher than TG2-L ones when compared with Aβ(1-42) exposed cells and with the controls. No significant change in TG2-L and TG2-S expression levels in cultures exposed to Aβ(35-25) was found. When OECs were exposed to 100 μM of indicaxanthin, a significant increase in both isoforms was found when compared with the controls. The pre-treatment of the cells with indicaxanthin and Aβ(1-42) exposure induced a significant increase of TG2-L when compared with TG2-S and with the controls. In contrast, indicaxanthin pre-treatment in Aβ(25-35) exposed cells caused a significant enhancement of TG2-S expression levels, when compared with TG2-L ones. Furthermore, no significant changes in TG2-L expression levels in Aβ(35-25) exposed OECs were observed. Surprisingly, indicaxanthin pre-treatment caused a significant increase in the TG2-S expression levels when compared with Aβ(35-25), indicaxanthin alone treated cells, and with the controls. Densitometric analysis performed for each experimental condition, after normalization with β-tubulin, confirmed all the results. These data highlighted that Aβ treatment both in the absence and presence of indicaxanthin differently modulates TG2 isoforms acting or on apoptotic pathway activation or on the cell self-renewal ability.

### 2.6. Caspase-3 Cleavage Immunolabeling

**Figure 8:**
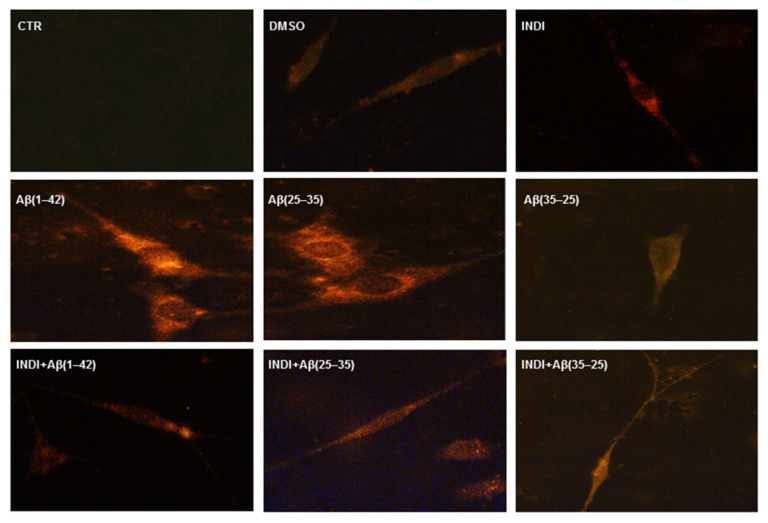
Immunocytochemistry for anti-caspase-3 in OECs. Images show different conditions: control, DMSO, 100 µM indicaxanthin (INDI), 10 μM Aβ(1-42) or Aβ(25-35) or Aβ(35-25) both in the absence and presence of 100 µM INDI for 24 h. Scale bar 20 µm.

To verify the TG2-mediated apoptotic pathway in OECs exposed to Aβ(1-42) or Aβ(25-35), we evaluated the caspase-3 cleavage through immunocytochemical techniques. Figure 8 highlights caspase-3-positive OECs exposed to different conditions. In the controls (PBS and DMSO), the positivity of cells for caspase-3 was almost absent. When the cells were exposed to Aβ(1-42) or Aβ(25-35), strong activation of positive cells for caspase-3 was found, which appeared mainly localized in the cytoplasm. The effect was particularly evident in Aβ(25-35), which is highly toxic for the cells. It is evident in Aβ(1-42 and 25-35) exposure of an increase in cell size. In Aβ(35-25)-treated cells, a light positivity for caspase-3, when compared with the controls, was found. The treatment of OECs with 100 μM of indicaxanthin did not produce any positivity for caspase-3, when compared with the controls. In indicaxanthin pre-treated cells and subsequently exposed to Aβ(1-42) or Aβ(25-35), a decrease for caspase-3-positive cells, which appeared at similar expression levels of the controls, was observed. A slight increase of positive OECs was found in indicaxanthin pre-treated cells and then exposed to the fragment Aβ(35-25) when compared with indicaxanthin alone and with the controls. No specific staining of OECs was observed in the control incubation in which the primary antibody was omitted. These findings revealed the role played by TG2 in the control of apoptotic pathway activation in both Aβ exposed OECs and indicaxanthin pre-treated ones.

### 2.7. Cyclin d_1_ Expression Levels and Nestin Immunolabeling

**Figure 9:**
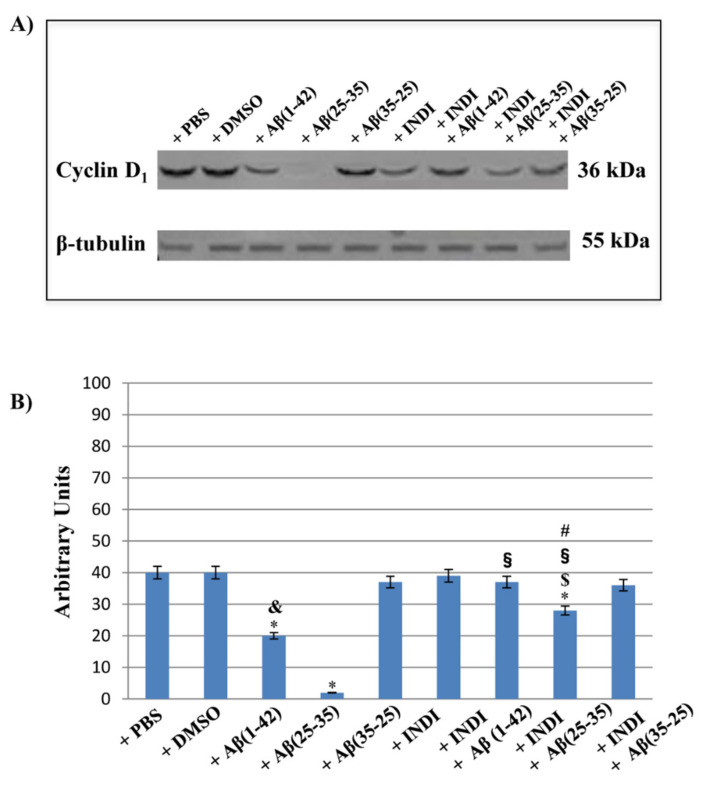
Western blotting analysis. (**A**) Representative immunoblot through western blotting analysis for cyclin D_1_ expression levels in total cellular lysates from OECs in different conditions: control, DMSO, 100 µM indicaxanthin, 10 μM Aβ(1-42) or Aβ(25-35) or Aβ(35-25) both in the absence and presence of 100 µM indicaxanthin for 24 h. (**B**) Densitometric analysis of cyclin d_1_ expression levels performed after normalization with β-tubulin. The results are expressed as the mean ± S.D. of the values of five separate experiments performed in triplicate. * *p* < 0.05 significant differences vs. controls. **^$^** *p* < 0.05 significant differences of Aβ(1-42) + INDI or Aβ(25-35) + INDI or Aβ(35-25) + INDI vs. INDI; **^§^** *p* < 0.05 significant differences of Aβ(1-42) + INDI vs. Aβ(1-42), Aβ(25-35) + INDI vs. Aβ(25-35) or Aβ(35-25) + INDI vs. Aβ(35-25); **^&^** *p* < 0.05 significant differences of Aβ(1-42) vs. Aβ(25-35 ) or vs. Aβ(35-25); **^#^** *p* < 0.05 significant differences of Aβ(25-35) + INDI vs. Aβ(1-42) + INDI or vs. Aβ(35-25) + INDI.

To assess the role played of TG2 in the cellular repair induced by indicaxanthin on OEC exposed to Aβ, cyclin D_1_ expression levels and the cell positivity for Nestin were examined. Western blot and densitometric analysis for cyclin d_1_ performed on total cellular lysates from Aβ(1-42), Aβ(25-35) and Aβ(35-25) exposed OECs both in the absence and presence of 100 μM indicaxanthin are reported in Figure 9A,B. A significant decrease in cyclin d_1_ expression levels in Aβ(1-42) exposed cells, when compared with the controls, were found. The toxic fragment Aβ(25-35) induced a very strong reduction of cyclin D_1_ expression levels compared to the full native Aβ(1-42) peptide and the controls. In contrast, the exposure of OECs to no-toxic fragment Aβ(35-25) did not cause significant modifications in cyclin d_1_ expression levels when compared with the controls. The pre-treatment of the cells with Indicaxanthin alone did not induce any significant change of protein expression levels, when compared with the controls. When it was added to OECs and following exposure to Aβ(1-42), a significant increase of cyclin D_1_ expression levels, when compared with Aβ(1-42) alone, was observed, and protein levels appeared similar to those found in the controls and indicaxanthin exposed cells alone. A significant increase in cyclin D_1_ expression levels in Aβ(25-35) treated with indicaxanthin was highlighted, when compared with Aβ(25-35) exposed cells (Figure 9A,B), even if its levels were lower than observed in cells treated with indicaxanthin plus Aβ(1-42), indicaxanthin alone, and in the controls.

**Figure 10:**
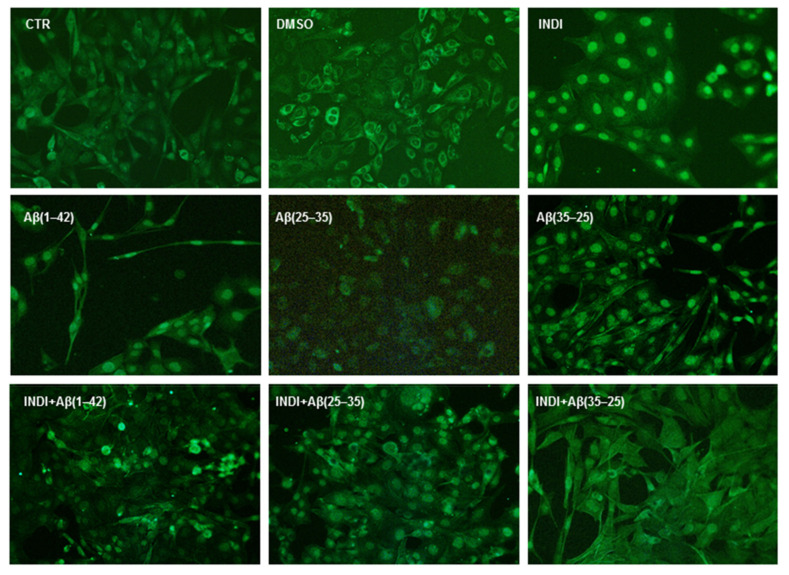
Immunocytochemistry for anti Nestin in OECs. Images show different conditions: control, DMSO, 100 µM Indicaxanthin (INDI), 10 μM Aβ(1-42) or Aβ(25-35) or Aβ(35-25) both in the absence and presence of 100 µM INDI for 24 h. Scale bar 20 µm.

Figure 10 reports the positivity for Nestin in OECs exposed to indicaxanthin in the absence and presence of Aβ(1-42), Aβ(25-35), and Aβ(35-25). A significant reduction in the number of positive cells for Nestin in Aβ(1-42) and Aβ(25-35) exposed was found when compared with the controls. Aβ(35-25) exposed cells showed a slight increase for Nestin positive OECs when compared with the controls. The pre-treatment of cultures with 100 μM of indicaxanthin for 24 h did not induce changes for Nestin positive cells, which appeared similar to the levels observed in the controls. When OECs were pre-treated with 100 μM of indicaxanthin and subsequently exposed to Aβ(1-42) or Aβ(25-35), a strong increase in positive cells for Nestin was found when compared with the controls. A low Nestin positivity in indicaxanthin pre-treated cells and then exposed to Aβ(35-35) was shown. No specific staining of OECs was observed in control incubation in which the primary antibody was omitted.

This set of experiments demonstrated that indicaxanthin pre-treatment stimulated TG2 repair activity in OEC exposed to Aβ, also activating the stem self-renewal through the increase of cyclin D_1_ expression levels and the cell positivity for Nestin.

## 3. Discussion

This study aimed to assess TG2 and its isoform expression levels in both OECs exposed to the full native peptide of Aβ(1-42) and its toxic fragment Aβ(25-35). Epidemiological evidence report that the effects of the Mediterranean Diet “MeDi” could be an alternative prophylaxis treatment for AD [27]. In particular, in Sicily, an increased frequency of centenarians has been identified, along with a reduced occurrence of mental and cognitive diseases when compared with other Italian or European regions [19,20]. One of the factors that could contribute to this phenomenon is the large availability of some rare specific nutrients, largely present in some areas of Sicily as well as indicaxanthin from *Opuntia ficus-indica* fruit. Therefore, for the first time, we tested the effect of indicaxanthin pre-treatment on OECs exposed to Aβ. Since the cytoskeleton plays an important role in the pathogenesis of neurodegenerative diseases including AD [28], particular attention was focused on the effect of indicaxanthin on some cytoskeletal proteins such as Vimentin and GFAP, which have an important role in astrogliosis, a typical sign of AD [29]. Furthermore, the expression levels of cyclin D_1_, which is induced in stem cell reprogramming and is co-expressed with Nestin, a marker of neural stem cells [26], were assessed. In addition, the effect of Aβ(1-42), Aβ(25-35), and Aβ(35-25) in the absence and presence of indicaxanthin was tested on cellular viability and on the activation of the apoptotic pathway. Intracellular total ROS and O_2_^−^ production was also evaluated. The experiments were performed on OECs because they represent a glial population of the olfactory system that is also involved in AD [12]. It is noted that olfactory dysfunction as well as hyposmia and olfactory memory loss represent the early symptoms of AD [18,30,31]. Furthermore, it has been demonstrated that the anterior olfactory nucleus (AON) projects to the hippocampus [32] and that it is the earliest site involved in AD, associated with cell loss, the neurofibrillary tangles, and senile plaques [12].

Our previous studies have demonstrated that TG2 is overexpressed in OECs exposed to Aβ(1-42) and its toxic fragment Aβ(25-35) and that the treatment with some growth factors (GFs) was able to restore its levels to control values [18]. In particular, TG2, a calcium-dependent protein with transamidanting activity, is involved in AD, inducing the formation of insoluble amyloid aggregates that can alter the properties of several proteins [2]. TG2 activity is downregulated in response to oxidative stress [33,34] and this effect could be related to the increase in the intracellular Ca^2+^ levels due to Aβ toxicity [18]. In fact, the accumulation of extracellular protein aggregates prevalently constituted by polymeric Aβ, caused by the aberrant transamidanting activity of TG2, are also related to a dysregulation of the autophagy process [35]. These conditions contribute to oxidative stress and neural cell death, in which TG2 plays a key role [34]. It has been reported that hippocampal neurons are more responsive to indicaxanthin [36]. In particular, it has an important role in several metabolic functions both in vitro and in vivo, reducing inflammation and enhancing immune response [22,23,37].

In this study, for the first time, we highlight that the OEC exposure to Aβ(1-42), its fragments Aβ(25-35) and Aβ(35-25) induce a different expression pattern of TG2-L and TG2-S, demonstrating the opposite role played by TG2. Furthermore, we show the protective effect exerted by indicaxanthin pre-treatment on total TG2 and its isoform expression levels. In particular, we found that in Aβ(1-42) treated cells, the two isoforms appeared at the same expression levels, whereas in the Aβ(25-35) exposed ones, TG2-S had higher levels than TG2-L when compared with Aβ(1-42) exposed cells and with the controls. In OECs exposed to Aβ(35-25), a slight modification between TG2-L and TG2-S expression levels was observed. The pre-treatment with indicaxanthin was able to counteract the oxidative damage following the exposure of the cells to the full native peptide of Aβ(1-42) and its toxic fragment Aβ(25-35), restoring the expression levels of total TG2 to the control values. Furthermore, CLSM analysis performed on single-cells showed that TG2 in OECs pre-treated with indicaxanthin alone was localized in the cytosol. In contrast, when cells were pre-treated with Indicaxanthin and then exposed to Aβ(1-42), the protein appeared prevalently localized into the nuclear compartment. In the cells pre-treated with indicaxanthin and then stressed with Aβ(25-35), TG2 was localized both in the cytosol and in the nucleus. Western blot analysis showed a significant increase in TG2-L in Indicaxanthin alone treated cells and in those then exposed cells to Aβ(1-42). This effect might be correlated to the role played by TG2 when it is localized into the nuclear compartment, in which it acts on the control of cell proliferation, regulating gene expression, cell survival and differentiation, exerting an anti-apoptotic function [10,18,38]. In OECs treated with indicaxanthin alone and in those subsequently exposed to Aβ(25-35), an increase of TG2-S expression levels was observed. The effect appeared more evident in the cells pre-treated with indicaxanthin. TG2-S, even if reduced compared to that found in Aβ(25-35) treated cells, exerts transamidanting activity and acts as an apoptotic factor [6,18]. Surprisingly, indicaxanthin pre-treatment in Aβ(35-25) exposed cells, induced a significant increase of TG2-S expression levels, when compared with Aβ(35-25) alone and controls. This finding might be due to the strong protective effect of indicaxanthin, since we hypothesize that Aβ(35-25) fragment, even if it was reported that is not toxic [25], was able to induce low toxicity in OECs, as relieved by a very significant increase of TG2-S expression levels when compared with exposed cells to Aβ(35-25) alone. Thus, we suppose that this effect may be due to the protective role played by TG2, which stimulates its pro-apoptotic activity, in order to remove damaged cells and to induce cellular repair. We also found that indicaxanthin counteracted the oxidative stress induced by Aβ, as relieved by the reduction of total ROS and O_2_^−^ production, which appeared similar to those observed in the controls. Thus, indicaxanthin pre-treatment, for its antioxidant properties, was able to reduce the Aβ-toxicity, oxidative stress-dependent and mitochondrial damage. In addition, indicaxanthin, with its anti-inflammatory proprieties, decreased GFAP and Vimentin expression levels, that were enhanced in Aβ exposed OECs. These results highlighted that indicaxanthin exerted a protective effect on reactive astrogliosis induced by Aβ, which is responsible of cytoskeleton modifications. Furthermore, to clarify the protective role played by TG2 in the absence and in the presence of indicaxanthin, the levels of Nestin, a marker of neural stem self-renewal, co-expressed with cyclin d_1_, a marker of cellular proliferation [26], were assessed. These results show an increase of positive cells for Nestin and cyclin d_1_ expression levels, demonstrating that indicaxanthin pre-treatment, stimulating the activity played by nuclear TG2 on stem self-renewal OEC reprogramming, that stimulates cell proliferation repairing the damage induced by Aβ. We also observed that indicaxanthin counteracted the TG2-aberrant cross-linking activity induced by Aβ-exposure on the cells, evaluating caspase-3 cleavage, that appeared reduced following its treatment. This effect might be correlated to the function that TG2 exerts on the apoptotic pathway, as revealed by the increase of TG2-S expression levels observed in our experimental conditions, when cells were treated with Aβ(1-42) and Aβ(25-35) in the absence of indicaxathin. In contrast, total TG2 did not show its opposite role on the basis of cellular localization and did not evidence the effect of Aβ both in the absence and in the presence of indicaxanthin.

**Figure 11:**
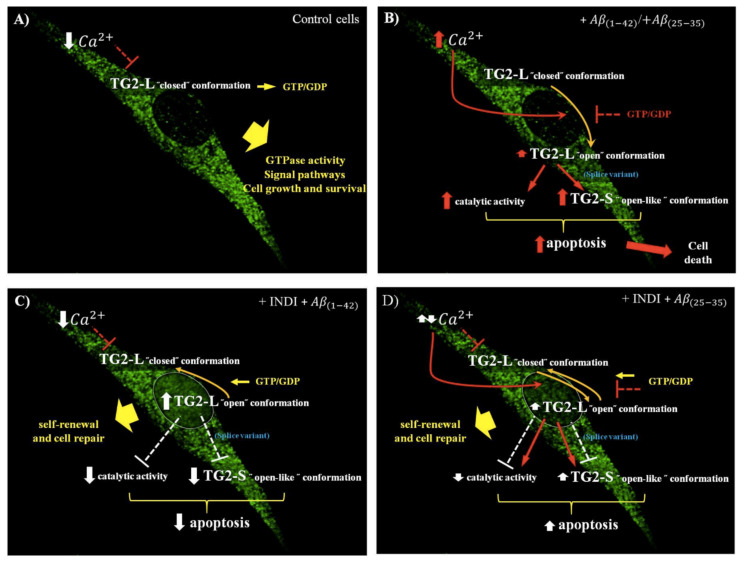
Drawing regarding the effects of OEC treatments with full native peptide Aβ(1-42) and Aβ(25-35) fragments on TG2 and its isoform (TG2-L and TG2-S) expression levels both in the absence and presence of indicaxanthin (INDI). (**A**) TG2-L and TG2-S expression levels in OECs maintained in normal conditions; (**B**) TG2-L and TG2-S expression levels in OECs exposed to 10 μM Aβ(1-42) or Aβ(25-35) for 24 h; (**C**) TG2-L and TG2-S expression levels in OECs pre-treated with 100 µM of INDI and exposed to 10 μM Aβ(1-42) for 24 h; (**D**) TG2-L and TG2-S expression levels in OECs pre-treated with 100 μM of INDI and exposed to 10 μM Aβ(25-35) for 24 h. Abbreviations: guanosine triphosphate (GTP); guanosine diphosphate (GDP). Color arrows: damage (red); partial recovery (yellow); no damage (white).

Taken together, our findings demonstrate that Aβ stress is responsible for TG2 upregulation [18,39] and its structural modifications in two distinct conformational states with different functions [10]. In fact, when the levels of Ca^2+^ are low and those of guanosine triphosphate (GTP) or guanosine diphosphate (GDP) are high, TG2-L acts as a GTPase, is involved in signaling pathway, is inactive, and is present in “closed” conformation, promoting cell growth and survival (Figure 11A). Aβ exposure of OECs, increasing intracellular Ca^2+^ and decreasing GTP or GDP levels, might cause a change of TG2-L from “closed” to “open” conformation, catalytically active. In addition, Aβ treatment induced an increase in the levels of TG2-S, an alternative splice variant of TG2 lacking the portion of the carboxyl-terminal essential for the maintenance of the protein in the “closed” conformation that is responsible for apoptotic activation and cell death. The effect is more evident when the cells were exposed to the major toxic Aβ(25-35) fragment, which strongly enhanced intracellular Ca^2+^ levels (Figure 11B). Furthermore, the increased expression levels of TG2-S following the exposure of OECs to Aβ(1-42) and Aβ(25-35) were accompanied by the activation of the apoptotic pathway, as revealed by caspase-3 cleavage. The effect was particularly evident with the toxic fragment Aβ(25-35). This finding is in agreement with other observations reporting that TG2-S was found in the brain of AD patients, and it also represents the prevalent isoform responsible of cell death [9]. Furthermore, the apoptotic activity of TG2-S is related or an excess of transamidanting activity, which possesses a weak ability to bind GTP and is not able to regulate the enzymatic activity of TG2, or to the capacity of TG2-S to lead cell aggregation [10]. Therefore, Aβ induced cell death in OECs, increasing the expression levels of TG2-S. The effect was also accompanied by the decrease of Nestin and cyclin d_1_ levels, and it was more evident in Aβ(25-35) exposed OECs, in which cyclin d_1_ disappears. Indicaxanthin pre-treatment prevented total TG2 overexpression induced by the OEC exposure to the full native peptide Aβ(1-42) and Aβ(25-35) fragment, probably binding to Ca^2+^ [37]. The significant increase in TG2-L isoform expression levels induced by Aβ(1-42), accompanied by the decrease in TG2-S ones, is related to the role that the protein plays into the nucleus, in which it might stimulate OEC self-renewal and the reparative effect against Aβ toxicity (Figure 11C). Furthermore, in Aβ(25-35) exposed OECs, indicaxanthin is able to significantly decrease TG2-S isoform expression levels, enhancing at the same time those of TG2-L. This effect was accompanied by the reduction of oxidative stress, caspase-3 cleavage, and in parallel by an increase in both the cyclin D1 and Nestin levels. The different expression pattern of TG2 isoforms in Aβ(25-35) exposed cells in the presence of indicaxanthin might be due to the major toxicity of the fragment, which induces a major enhancement of Ca^2+^. Thus, in these conditions, the protein was able to stimulate both apoptosis and self-renewal (Figure 11D).

## 4. Materials and Methods

### 4.1. Materials

Leupeptin, aprotinin, phenylmethylsulfonyl fluoride (PMSF), EDTA, EGTA, sodium dodecyl sulfate (SDS), phosphatase inhibitor cocktail II, cytosine arabinoside, full native peptide of Aβ(1-42), fragment Aβ(25-35), fragment Aβ(35-25), 3(4,5-dimethyl-thiazol-2-yl)2,5-diphenyl-tetrazolium bromide (MTT), dimethyl sulfoxide (DMSO), Lab-Tek II Chamber-Slide Systems, paraformaldehyde, and other analytical chemicals were purchased from Sigma-Aldrich (Milan, Italy). Acetic acid and methanol were of LC grade and purchased from Merck (Milan, Italy). Trypsin, antibiotics, heat inactivated fetal bovine serum (GIBCO), phosphate buffer saline solution (PBS), normal goat serum (NGS, GIBCO), modified Eagle medium (MEM) with 2 mM GlutaMAX (GIBCO), nitrocellulose membrane filter paper sandwich 0.45 µm pore size (Invitrogen), mouse monoclonal antibody against β-tubulin, anti-rabbit IgG horseradish peroxidase–conjugated, and anti-mouse IgG horseradish peroxidase–conjugated were from Thermo Fisher Scientific (Milan, Italy). Bicinconinic acid method (Pierce/Thermo-Scientific, Rockford, IL, USA). Mini-PROTEAN^®^ TGX™ Precast Protein Gels (4–15%), Mini Trans Blots Filter Paper, 10× Tris/Glycine/SDS buffer, 10× Tris/Glycine buffer, 4× Laemmli Sample Buffer, 2-mercaptoethanol, Precision Plus Protein^TM^ Standard Dual Color, were from Bio-Rad Laboratories Srl (Milan, Italy). Rabbit monoclonal antibody against cyclin D_1_ was from Millipore (Milan, Italy). Mouse monoclonal antibody against TG2 (neomarkers), mouse monoclonal antibody against Nestin and cellular ROS/superoxide detection assay were from Abcam (Milan, Italy). Mouse monoclonal antibody against GFAP and mouse monoclonal antibody against Vimentin were from DAKO. Mouse monoclonal antibody against caspase-3 was from Becton-Dickinson (Milan, Italy). Cy3 goat anti-mouse and fluorescein isothiocyanate (FITC)-conjugated goat anti-mouse IgG antibody were from Jackson Immunological Research Laboratories Inc. Western Lightning Plus-ECL enhanced chemiluminescence substrate was from Perkin-Helmer (Monza, Italy).

### 4.2. Animals

Experiments were carried out on 2-day-old mouse pups (P2) provided by Envigo RMS s.r.l. (Italy). Animals were kept in a controlled environment (23 ± 1 °C, 50 ± 5% humidity) with a 12 h light⁄dark cycle with food and water available ad libitum. Experiments were carried out in compliance with the Italian law on animal care no. 116/1992 and no. 26/2014 and in accordance with the European Community Council Directive (86/609/EEC) and were approved (authorization no. 174/2017-PR) by the Ethical Committee at the University of Catania (Italy). Efforts were made to minimize the number of animals used.

### 4.3. Olfactory Ensheathing Cell (OEC) Cultures

Olfactory bulbs were removed from decapitated pups and placed in cold (+4 °C) Leibowitz L-15 medium [40]. Successively, pellets were digested in MEM-H, containing collagenase and trypsin mixture. Trypsinization was stopped by adding DMEM supplemented with 10% FBS (DMEM/FBS). Cells were re-suspended and plated in flasks fed with complete DMEM/FBS. Cytosine arabinoside (10^−5^ M), an antimitotic agent, was added 24 h after initial plating, in order to reduce the number of dividing fibroblasts. OECs were then processed to an additional step transferring from one flask to a new one, in order to reduce contaminating cells, following the method by Chuah and Au [41]. When OECs were confluent, they were removed by trypsin, transferred in 25 cm^2^ flasks, and cultured in DMEM/FBS. Cells were then incubated at 37 °C in complete medium and fed twice a week. Purified OECs were grown in DMEM/FBS on 14 mm diameter glass coverslips and 96-multi-wells flat bottomed at a final density of 1 × 10^4^ cells/coverslip and in 25 cm^2^ flasks at a final density of 1 × 10^6^. Cells were then incubated at 37 °C in a humidified 5% CO_2_–95% air mixture.

### 4.4. Indicaxanthin Purification

Indicaxanthin was isolated from *Opuntia ficus indica* L. Mill fruit pulp (yellow cultivar) and purity (97%) of the pigment was assessed by HPLC, according to a previously described method [24].

### 4.5. Treatment of OECs

OECs were divided into different groups: a group was stressed for 24 h with of 10 μM Aβ(1-42) or Aβ(25-35) or Aβ(35-25) [18]; another group was treated with 50 µM or 100 µM of indicaxanthin for 24 h; the other two groups were pre-treated with 50 µM or 100 µM of indicaxanthin for 30 min and subsequently were exposed to 10 μM Aβ(1-42) or Aβ(25-35) or Aβ(35-25). Stock solutions of full native peptide Aβ(1-42), Aβ(25-35) and Aβ(35-25) fragments were diluted in DMSO. For every test, the suitable aliquot from each stock solution was added to the culture medium, in order to obtain a final concentration 10 µM. A group of cells was treated with a corresponding volume of PBS (final concentration 0.01% *v*/*v*) and used as the control. Another group of cell cultures was treated with the corresponding volume of DMSO used to solubilize full native peptide Aβ(1-42) and Aβ fragments, having a final DMSO concentration of 0.01% *v*/*v*.

### 4.6. 3(4,5-dimethyl-thiazol-2-yl)2,5-diphenyl-tetrazolium Bromide (MTT) Bioassay

In untreated and treated OECs, 20 μL of 0.5% MTT solution were added to each multiwell as previously reported [18]. After 2 h of incubation at 37 °C, the supernatant was removed, replaced with 200 μL DMSO, and incubated at 37 °C for 1 h. The optical density of each well sample was measured with a microplate spectrophotometer reader (Titertek Multiskan; Flow Laboratories, Helsinki, Finland) at λ = 570 nm. Based on the MTT test, we chose the optimal concentration of indicaxanthin to treat OECs both in the absence and presence of 10 μM Aβ(1-42), Aβ(25-35) Aβ(25-35), 100 µM for 24 h. To exclude eventual interference of indicaxanthin with the MTT solution, several washes with PBS in the untreated and treated cells both in the absence and presence of indicaxanthin were performed and the absorbance of lysed cells was evaluated at λ = 570 nm. No significant differences between the absorbances at λ = 570 nm in the control and indicaxanthin-treated cells were observed. Cell viability (%) was expressed as a percentage relative to the untreated cell one (PBS; controls), which were assumed as 100% of cell viability.

### 4.7. Total ROS/O_2_^−^ Production

In untreated and treated OECs, total ROS and O_2_^−^ production was assessed through the Cellular ROS/Superoxide Detection Assay, according to the manufacturer’s instruction. The fluorescent products generated by the two dyes were green for total intracellular ROS and orange for O_2_^−^ detection were visualized using a wide-field Zeiss fluorescent microscope (Zeiss, Germany) equipped with standard green (λ_Ex_/λ_Em_ = 490/525 nm) and orange (λ_Ex_/λ_Em_ = 550/620 nm) filter set.

### 4.8. Immunocytochemical Technique and Confocal Laser Scanning Microscope (CLSM) Analysis

To assess the positivity for Vimentin (proliferation marker), GFAP (differentiation marker), caspase-3 (apoptotic marker), and TG2 in untreated and treated, OECs were processed through immunocytochemical procedures. After 24 h, all cells were fixed through 4% paraformaldehyde in 0.1 M PBS for 30 min and then incubated overnight at 4 °C in the following primary antibodies: mouse monoclonal antibody against Vimentin (1:50), mouse monoclonal antibody against GFAP (diluted 1:1000), mouse monoclonal antibody against Nestin (1:200), mouse monoclonal antibody against Caspase-3 (1:500), and mouse monoclonal antibody against TG2 (1:200). FITC anti-mouse (diluted 1:200) and Cy3 anti-mouse (diluted 1:500) were used as secondary antibodies for 1 h at room temperature and in dark condition. Successively, coverslips were washed in PBS and mounted with PBS/glycerol. The immunostained coverslips were analyzed on a Zeiss fluorescent microscope (Zeiss, Germany) and images were captured with an Axiovision Imaging System for GFAP, Nestin, Vimentin, and caspase-3. The immunostained for TG2 was obtained using a confocal laser scanning microscope (CLSM) 510 Meta (Zeiss, Germany), using a X63 lens and captured with an Axiovision Imaging System [6,18,33]. The positive labeled cells were counted in ten different microscopic fields (20× magnification) and the positivity for each marker was expressed as a percentage and compared with each respective control. No non-specific staining of OECs was observed in control incubations in which the primary antibodies were omitted. To analyze TG2 positive cells, confocal laser scanning microscope (CLSM, LSM-510 Meta, Zeiss, Germany) was used. For the acquisition with CLSM, we used an Apo 63 ×/1.4 oil immersion objective and the argon (λ = 488 nm) and HeNe (λ = 543 nm) lasers. Images were acquired at the pixel resolution of 1024 × 1024 and were processed to enhance brightness and contrast using the software ZEN 2009. The version number for software ZEN 2009 was 5.5.0.452 and provided together ZEISS confocal microscope. The ZEN 2009 soft version is available at https://www.softpedia.com/get/Multimedia/Graphic/Graphic-Viewers/ZEN-2009-Light Edition.shtml (Accessed on 24 April 2013). The optical fields were examined through green fluorophore excitation.

### 4.9. Isolation of Total Protein and Western Blot Analysis

Untreated and treated OECs were harvested in cold PBS, collected by centrifugation, resuspended in cell lysis buffer containing 50 mM Tris-HCl (pH 6.8), 150 mM NaCl, 1 mM EDTA, 0.1 mM PMSF, 10 µg/mL of aprotinin, leupeptin, pepstatin, incubated for 30 min at 4 °C, centrifuged at 12,000× *g* for 10 min at 4 °C, and the supernatants containing total cell proteins were collected [5,18,34,42]. Briefly, extracted proteins were stored at −80 °C, and protein quantitation was performed by the bicinchoninic acid method, according to the manufacturer’s instruction. A total of 40 µg of total proteins were separated through 4–15% precast SDS–polyacrylamide gels and transferred to nitrocellulose membranes. Filters obtained were then incubated with the following 1:1000 diluted antibodies: mouse monoclonal antibody against TG2, rabbit monoclonal antibody against Cyclin d_1_, mouse monoclonal antibody against β-tubulin. Anti-rabbit IgG horseradish peroxidase–conjugated and anti-mouse IgG horseradish peroxidase-conjugated were then used. The expression of each protein was visualized through Western Lightning Plus-ECL enhanced chemiluminescence substrate after autoradiography filter exposure. Blots were then scanned and quantified through ChemiDoc Imaging System (ChemiDoc™ Imaging System, Bio-Rad, Milan, Italy). Densitometric analysis was performed through the integrated software and data obtained were normalized with β-tubulin.

### 4.10. Statistical Analysis

Data were statistically analyzed using one-way analysis of variance (one-way ANOVA) followed by the post-hoc Holm–Sidak test to calculate significant differences among groups. Reported data represent the mean ± S.D. of five separated experiments in triplicate, and differences among groups were considered to be significant at * *p* < 0.05.

## 5. Conclusions

Our findings clearly highlighted that Aβ exposure on OECs induced an increase in TG2 and a different expression pattern of its isoforms. Furthermore, the pre-treatment of the cells with indicaxanthin was able to decrease the total TG2 expression levels, inducing a different pattern of TG2 isoforms that might be due to a change in TG2 state conformation. It also reduced total ROS and O_2_^−^ production and the expression levels of GFAP and Vimentin, inhibiting glial reactivity and the activation of the apoptotic pathway induced by Aβ. Furthermore, it leads to an increase in Nestin and cyclin D_1_ expression levels, stimulating OEC self-renewal and the TG2 reparative role. In addition, our data suggest that OECs exposed to Aβ both in the absence and presence of indicaxanthin might differently induce the transition of TG2 between “closed” and “open” conformation, providing a new mechanism involved in the signal pathways activated by the protein in Aβ injury. Therefore, further studies need to better clarify whether indicaxanthin plays an important role in the adoption of the TG2 open conformation, which has a key role in the self-renewal ability of OECs, being cells capable of expressing and releasing neurotrophic receptors. As a consequence, it might represent a promising tool for neural regeneration in AD.

## Data Availability

The data used and analyzed during the current study are available from the corresponding author on reasonable request.

## Abbreviations

AD	Alzheimer’s disease
Aβ	Amyloid Beta
TG2	Tissue transglutaminase
TG2-L	Tissue transglutaminase Long
TG2-S	Tissue transglutaminase Short
OECs	Olfactory Ensheathing Cells
ROS	Reactive Oxygen Species
O2−	Superoxide anion
GFAP	Glial Fibrillary Acidic Protein
DMEM	Dulbecco Modified Eagles Medium
FBS	Fetal Bovine Serum
